# Fitness checklist model for spontaneous breathing tests in pediatrics

**DOI:** 10.5935/2965-2774.20230312-en

**Published:** 2023

**Authors:** Bruno Silva Miranda, Valéria Cabral Neves, Yessa do Prado Albuquerque, Emilly Freitas de Souza, Adriana Koliski, Mônica Nunes Lima Cat, José Eduardo Carreiro

**Affiliations:** 1 Complexo do Hospital de Clínicas, Faculdade de Medicina, Universidade Federal do Paraná - Curitiba (PR), Brazil

**Keywords:** Airway extubation, Respiration, artificial, Checklist, Respiratory function test, Child, Intensive care units, pediatric

## Abstract

**Objective:**

To evaluate whether a model of a daily fitness checklist for spontaneous breathing tests is able to identify predictive variables of extubation failure in pediatric patients admitted to a Brazilian intensive care unit.

**Methods:**

This was a single-center, cross-sectional study with prospective data collection. The checklist model comprised 20 items and was applied to assess the ability to perform spontaneous breathing tests.

**Results:**

The sample consisted of 126 pediatric patients (85 males (67.5%)) on invasive mechanical ventilation, for whom 1,217 daily assessments were applied at the bedside. The weighted total score of the prediction model showed the highest discriminatory power for the spontaneous breathing test, with sensitivity and specificity indices for fitness failure of 89.7% or success of 84.6%. The cutoff point suggested by the checklist was 8, with a probability of extubation failure less than 5%. Failure increased progressively with increasing score, with a maximum probability of predicting extubation failure of 85%.

**Conclusion:**

The extubation failure rate with the use of this model was within what is acceptable in the literature. The daily checklist model for the spontaneous breathing test was able to identify predictive variables of failure in the extubation process in pediatric patients.

## INTRODUCTION

Weaning from invasive mechanical ventilation (IMV) is a critical period of transition from mechanical respiratory support to spontaneous respiratory control by the patient himself or herself.^(1,2)^ This process should be based on evaluations of the normality of clinical, radiological and laboratory parameters to avoid failure.^(3)^ Thus, it is extremely important to establish protocols for the safe application of IMV, including the assessment of readiness for elective extubation, with the objective of minimizing the morbidity associated with extubation failure and prolonged IMV.^(4)^

The spontaneous breathing test (SBT) is used to assess whether patients are fit for extubation and thus minimize the duration of IMV and decrease complications associated with hospitalization.^(4)^ For this purpose, the child is placed on minimum IMV settings for a certain time, when signs of respiratory distress and blood gas changes are evaluated.^(5,6)^ Some studies conducted in the pediatric population attempted to identify predictors of successful extubation but were unable to determine the exact set of parameters sufficient for this discrimination.^(5,6,7)^ Thus, there are no data in the literature pointing to the superiority between the types of SBT performance methods;^(8)^ therefore, the combined factors that may predict test failure or success remain uncertain.^(9,10,11)^

Checklists are usually used in the hospital environment as tools for systematic approaches to ensure the quality of care processes^(12)^ because they facilitate the interaction and integration of the professionals involved in their implementation.^(13)^ While some checklists have been published to monitor ventilatory weaning in the adult population,^(14,15,16)^ in the pediatric population, there is a lack of research on the subject.

Thus, the objective of this study was to evaluate whether a daily checklist model of fitness for SBT can identify predictive variables of failure in the extubation process in pediatric patients admitted to the pediatric ICU.

## METHODS

This study was approved by the Ethics Committee for Research on Human Beings of the institution (CAAE 91370818.0.0000.0096), and the results were presented according to the STrengthening the Reporting of OBservational studies in Epidemiology (STROBE) protocol.

This was a cross-sectional study conducted through the Graduate Program in Child and Adolescent Health, with data collection performed in the pediatric ICU of *Complexo do Hospital de Clínicas da Universidade Federal do Paraná* (UFPR) from August 2018 to August 2019, with the objective of internally validating a daily fitness checklist model for SBT.

Patients aged between 28 days and 14 years, with more than 24 hours and less than 30 days of IMV, with parental and/or guardian consent, and who signed the informed consent form were included in the study. Patients with tracheostomy, those who died prior to extubation, as well as those who withdrew informed consent were excluded.

**Figure 1:**
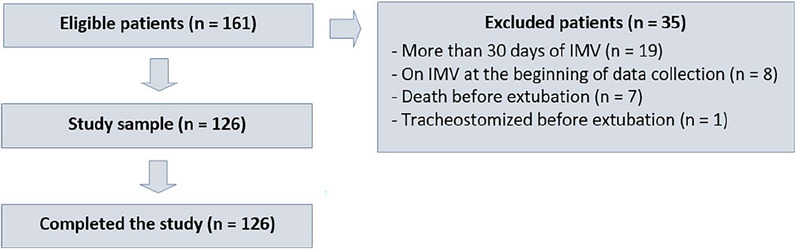
Flowchart of patient inclusion into the study

During the study period, 388 patients were hospitalized and underwent IMV, of whom 126 met the inclusion criteria (Figure 1).

IMV - invasive mechanical ventilation.

The researchers developed a daily fitness checklist model for SBT that consisted of 20 variables, scored as one when present in the daily assessment at the bedside and zero when absent, with the final score ranging from zero to 20 (Table 1).

**Table 1:** Variables of the daily fitness checklist for the spontaneous

**Mechanical ventilation**	1.PIP ≥ 20cmH_2_O
	2. PEEP ≥ 6cmH_2_0
	3. FiO_2_ ≥ 40%
	4. VT ≤ 6mL/kg
	5. RSBI ≥ 6.5rpm/minute/mL/kg
**Laboratory tests/imaging**	6. Abnormalities on chest radiography
	7. Important blood gas disorders
	8. PaO_2_/FiO_2_ ≤ 200mmHg
	9. Hb < 8g/dL
**Medications**	10. Use of vasoactive drugs
	11. Use of neuromuscular blockade in the last 24 hours
**Patient clinical factors**	12. Cause leading to tracheal intubation unresolved
	13. Hypersecretive patient
	14.SpO_2_ ≤ 90%
	15. RR altered for age
	16. HR altered for age
	17. BP altered for age
	18. Abdominal distension
	19. Absence of cough
	20. Positive water balance in the last 24 hours

PIP - positive inspiratory pressure; PEEP - positive end-expiratory pressure; FÍO_2_ - fraction of inspired oxygen; VT - tidal volume; RSBI - rapid and shallow breathing index; PaO_2_ - partial pressure of oxygen; Hb - hemoglobin; SpO_2_ - peripheral oxygen saturation; RR - respiratory rate; HR - heart rate; BP - blood pressure.

The 20 variables include in the daily SBT fitness checklist were studied using multivariate logistic regression to identify those variables with the greatest discriminatory power to better predict extubation failure and the risk of tracheal extubation failure, estimated by adding the scores for the variables, multiplied by their weights, divided by the sum of the weights

[math]

The discriminatory power and the predictive power for extubation failure of the generated scores were evaluated using receiver operating characteristic (ROC) curves and univariate logistic regression, respectively.

The checklist was applied in the morning by the physical therapy team to all patients until the decision of tracheal extubation. This decision was always made on the basis of institutional practice, through laboratory and imaging tests, without interference from the authors.

Patients were considered eligible for tracheal extubation and SBT on the basis of the unit’s routine: stable clinical data, chest X-ray and previous arterial blood gas analysis, with diet and sedoanalgesia suspended 3 hours before the procedure and prophylactic use of corticosteroids at least 30 minutes before the procedure to prevent upper airway obstruction.

All patients submitted to the SBT protocol were placed in pressure support ventilation mode for 30 minutes, with a value of 7cmH_2_O above the positive end-expiratory pressure (PEEP), with a PEEP of 5cmH_2_O and inspired oxygen fraction (FiO_2_) ≤ 40%. The level of consciousness was assessed every 10 minutes using the Glasgow coma scale; tidal volume was assessed using the mechanical ventilator; and heart, respiratory and blood pressure rates were monitored to ensure that they were within normal limits for each patient’s age group. When values were within the normal range, tracheal extubation was performed, recording posttracheal conditions such as the need for inhalation with adrenaline, respiratory support (oxygen therapy or noninvasive ventilation) or change in the state of consciousness and vital data. In the presence of any change during the 30-minute SBT, the previous IMV parameters were reinstated, with reassessment at 24 hours.

The primary outcome measure evaluated in the study was extubation failure, which can be defined as the inability to maintain spontaneous breathing in the first 48 hours after removal of the tracheal tube.

For data analysis, the Mann-Whitney test and the Pearson/Yates chi-square test were applied. A multivariate logistic regression model was applied to identify the variables with the highest prediction and their respective odds ratios (ORs). ROC curves were constructed to estimate the discriminant power of the independent variables for the indication of SBT and planned extubation and to establish the weights of the variables. A univariate logistic regression model was applied to identify the probability of SBT and planned extubation based on different scores and to establish the sensitivity, specificity and cutoff point. For all analyses, p < 0.05 was considered the minimum level of significance (Statistica 4.0, StatSoft Power Solutions, Inc., Palo Alto, California, USA).

The sample size to evaluate the accuracy of the daily fitness checklist for SBT was estimated with a sensitivity of 90%, type I error of 5% and margin of error of 5%, resulting in a suggested sample size of 126 participants.

## RESULTS

The study sample comprised 126 patients who followed the proposed protocol of clinical evaluation, SBT and extubation. For these patients, the indication for IMV was mainly lung disease; among the 17 patients with other causes of indication for intubation, 11 (8.7%) were patients with neurological diseases, 3 (2.3%) were patients with some hematological disease, and 3 (2.3%) were patients with endocrine disease complications (Table 2).

**Table 2:** Characteristics of the patients who were indicated for the spontaneous breathing test

Variables	
Sex	
Male	85 (67.5)
Female	41 (32.5)
Weight (kg)	11.0 [6 -20]
Age (months)	23.0 [7-59]
Infant	63 (50.0)
Preschool	30 (23.8)
School	19 (15.1)
Teenager	14 (11.1)
Endotracheal tube	125 (99.2)
Cause of tracheal intubation	
Lung disease	74 (58.7)
Postoperative	35 (27.7)
Other causes	17 (13.4)

Results expressed as n (%) or median [interquartile range].

A total of 1,217 daily checklist evaluations were performed, and the total score was significantly lower among the observations with indications of SBT [4.0 (3.0 - 5.0) *versus 9.0* (7.0 - 11.0), p < 0.001] (Table 3).

**Table 3:** Variables of the daily fitness checklist for the spontaneous breathing test in the groups of patients with and without indications for the spontaneous breathing test

Variables	nSBT (n = 1091)	wSBT (n = 126)	p value
Indication of unresolved intubation	991 (90.8)	27 (21.4)	< 0.001
Hypersecretive patient	835 (76.5)	74 (58.7)	< 0.001
Radiograph with changes	588 (53.9	15 (11.9)	< 0.001
PIP > 20mmHg	687 (63.0)	19 (15.1)	< 0.001
PEEP > 6cm/H_2_O	957 (87.7)	81 (64.3)	< 0.001
FiO_2_ > 40%	542 (49.7)	22 (17.5)	< 0.001
SpO_2_ < 90%	122 (11.2)	4 (3.2)	0.01
Altered respiratory rate	654 (59.9)	29 (23.0)	< 0.001
Altered heart rate	149 (13.7)	10 (7.9)	0.09
Altered blood pressure	184 (16.9)	9 (7.1)	< 0.01
VT < 6 - 8 mL	232 (21.3)	9 (7.1)	< 0.001
SBI > 6.5	688 (63.1)	57 (45.2)	< 0.001
Gasometric disorders	433 (39.7)	20 (15.9)	< 0.001
PaO_2_/FiO_2_ ratio < 200	418 (38.3)	14 (11.1)	< 0.001
Hemoglobin < 8g/dL	189 (17.3)	11 (8.7)	0.01
Positive water balance (24 hours)	431 (39.5)	36 (28.6)	0.02
Abdominal distension	382 (35.0)	28 (22.2)	< 0.01
Use of vasoactive drugs	517 (47.4)	22 (17.5)	< 0.001
Neuromuscular block (24 hours)	425 (39.0)	12 (9.5)	< 0.001
Absence of cough	454 (41.6)	9 (7.1)	< 0.001
Total %	9 (7-11)	4 (3 - 5)	< 0.001

nSBT - no spontaneous breathing test indicated; wSBT - with indication for the spontaneous breathing test; PIP - positive inspiratory pressure; PEEP - positive end-expiratory pressure; FiO_2_ - fraction of inspired oxygen; VT - tidal volume; SBI - shallow breathing index; PaO_2_ - partial pressure of arterial oxygen. Pearson/Yates chi-square test. The results are expressed as n (%).

In the multivariate logistic regression analysis to identify the variables with the highest discriminatory power for SBT, six were significant: indication of unresolved tracheal intubation, abnormalities on chest radiography, positive inspiratory pressure (PIP) ≥ 20mmHg, PEEP ≥ 6cm/H_2_O, ratio between partial pressure of oxygen (Pa02) and FiO_2_ ≤ 200 and absence of cough. These variables generated the so-called weighted total score (WTS), calculated using the weighted average equation, with attribution of the weights indicated by the OR (Table 4).

**Table 4:** Odds ratios and 95% confidence intervals for the variables of the daily fitness checklist for the spontaneous breathing test

Variables	Odds ratio	95%CI	p value
Indication of unresolved tracheal intubation	4.23	3.03-5.92	< 0.001
Hypersecretive patient	1.31	0.76-2.27	0.32
Radiograph with changes	4.52	2.80-7.30	< 0.001
PIP ≥ 20mmHg	4.17	2.75-6.33	< 0.001
PEEP ≥ 6cm/H20	1.36	1.19-1.55	< 0.001
FiO_2_ > 40%	1.20	0.62-2.31	0.57
SpaO_2_ < 90%	1.52	0.32-4.76	0.75
Altered respiratory rate	1.06	0.58-1.96	0.84
Altered heart rate	1.36	0.56-3.26	0.49
Altered blood pressure	2.43	0.95-6.66	0.05
VT < 6-8mL	1.75	0.70-4.54	0.21
SBI > 6.5	1.40	0.84-2.32	0.19
Gasometric disorders	1.08	0.55-2.12	0.82
PaO_2_/FiO_2_ ratio < 200	2.43	1.16-5.26	0.01
Hemoglobin < 8g/dL	2.29	0.95-5.52	0.07
Positive water balance (24 hours)	1.02	0.58-1.78	0.95
Abdominal distension	1.19	0.66-2.17	0.56
Use of vasoactive medications	1.03	0.53-2.00	0.93
Neuromuscular block (24 hours)	2.08	0.98-4.54	0.06
Absence of cough	5.82	3.09-10.97	< 0.001

95%CI - 95% confidence interval; PIP - positive inspiratory pressure; PEEP - positive end-expiratory pressure; FiO_2_ - fraction of inspired oxygen; SpaO_2_ - peripheral oxygen saturation; VT - tidal volume; SBI - shallow breathing index; PaO_2_ - partial pressure of arterial oxygen. Multivariate logistic regression.

Using the total score, the checklist model for SBT predicted tracheal extubation failure with a sensitivity of 83.3% and specificity of 86.7%, with a cutoff point of 5 points. However, with WTS, higher sensitivity (89.7%) was obtained, with a significantly lower number of variables and a cutoff of eight points (Table 5).

**Table 5:** Area under the curve, sensitivity, specificity, cutoff point and maximum probability of estimation of extubation failure for the scores of the daily fitness checklist for the spontaneous breathing test

Scores	AUC	Sensitivity	Specificity	Cutoff point	Maximum probability of estimate
Total	0.89	83.3	86.7	5	90
Total weighted	0.92	89.7	84.3	8	85

AUC - area under the curve.

**Figure 2:**
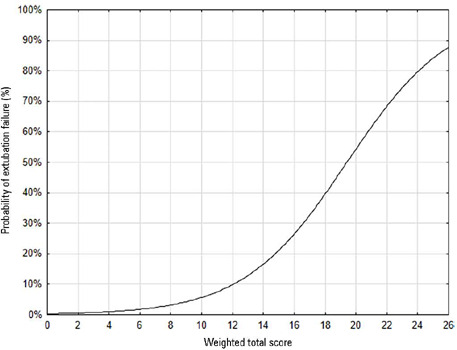
Probability of extubation failure based on the weighted total score of the daily fitness checklist for the spontaneous breathing test

The cutoff of eight points indicated by the ROC curve was the same as that indicated by the univariate logistic regression; when the probability of extubation failure was less than 5%, the score obtained progressively increased, with a maximum probability of predicting extubation failure of 85% with a score of 26 (Figure 2).

Univariate logistic regression: p < 0.001

The nine patients in whom extubation failed after SBT were reintubated (7.1%) due to altered level of consciousness (2), upper airway obstruction (3) and postextubation respiratory distress (4). Six patients received noninvasive ventilation (NIV), and three received oxygen therapy prior to reintubation. The median number of days in the ICU for patients who were reintubated was 11.0 (interquartile range - IQR 8.5 - 24.0).

## DISCUSSION

The checklist model for SBT, composed of 20 variables, was able to predict tracheal extubation failure with a sensitivity of 83.3% and specificity of 86.7%, with a cutoff point of 5 points. However, with WTS, a sensitivity of 89.7% was obtained for a weighted model with only six variables - indication of unresolved tracheal intubation, chest X-ray abnormalities, PIP ≥ 20 mmHg, PEEP ≥ 6cm/H_2_O, PaO_2_/FiO_2_ ≤ 200 and absence of cough, with a good predictive power for extubation failure and a cutoff point of 8 points.

Daily evaluations for extubation are considered an important practice for safe extubation in ICUs^(17,18)^ and strongly recommended in pediatric ventilator weaning.^(19)^

The transition from IMV to spontaneous breathing is complex and tests the functionality of multiple organs.^(20)^ Thus, to wean from a ventilator, the patient must be hemodynamically stable, and the cause that led to tracheal intubation must have resolved to ensure safe and successful extubation.^(21)^ An association between chest X-ray abnormalities and extubation failure has also been reported.^(22)^ Thus, it is inferred that ventilatory parameters may indicate the persistence of respiratory disease and, consequently, failure in the extubation process,^(10)^ justifying the use of parameters such as PIP, PEEP, and PaO_2_/FiO_2_ ≤ 200 as well as abnormalities on chest radiography.

Other authors have also highlighted the importance of evaluating cough during weaning and tracheal extubation.^(23)^ Evidence suggests a strong association between a weak or absent cough reflex and extubation failure in pediatric patients. ^(24,25,26)^

Extubation failure occurred in nine patients (7.1%) of the sample, a percentage similar to that found in the literature, i.e., between 5 and 12%.^(26,27)^ The percentage of extubation failure within the normal range can be explained by the protocol used, as standardization provides important information for the team to manage the extubation process, supporting the idea that SBT can be applied safely in a pediatric ICU.^(6)^ Another possible explanation is that SBT was conducted by a physiotherapist, confirming the idea that respiratory weaning therapists add an additional level of safety for pediatric patients without increasing IMV duration, length of hospital stay or extubation failure rate.^(28,29)^

There is no concensus on the prophylactic administration of corticosteroids prior to tracheal extubation, and its efficacy is still under debate.^(27,30)^ Therefore, even with prophylactic corticosteroids, three patients presented with upper airway obstruction. This condition is the major cause of reintubation in the pediatric population, so much so that airway evaluations such as the cuffleak test and the airway patency test are indicated.^(18)^ However, a negative test, which suggests the absence of leakage, should not delay an extubation attempt;^(8)^ therefore, this procedure was not considered in the checklist model proposed herein, although some researchers claim that it is a safe method for evaluating and preventing postextubation stridor.^(31,32,33)^

The creation of daily assessment protocols for pediatric ventilator weaning that identify the ideal time to perform SBT is essential to ensure extubation safety in these patients. However, the identification of risk factors associated with extubation failure still represents a challenge in pediatric ICUs. There is no consensus that indicates precisely which variables are important during the extubation process and which of those factors should receive more attention from evaluators. In this context, it is important for new daily assessment tools to be implemented, both in digital media and free platforms, to facilitate multiprofessional teams in the identification of patients able to be weaned from ventilation.

The model developed herein for predicting the risk of extubation failure was internally validated, meaning that it was developed and tested with the same sample, and ORs and regression coefficients were used to assign weights to variables with lower or higher predictive power, with a reduction in variables when using WTS. The results obtained should, therefore, be interpreted considering this limitation, and external validation studies, with the application of the daily fitness checklist for SBT in other samples, should be conducted to confirm its applicability Other limitations include the noninclusion of an airway patency test prior to extubation for patients at high risk of stridor and the unicentric nature of the study; therefore, the model should be applied with caution in other institutions due to institutional peculiarities.

## CONCLUSION

The checklist model analyzed for the spontaneous breathing test was able to predict tracheal extubation failure with good sensitivity and specificity, with unresolved tracheal intubation, chest X-ray abnormalities, positive inspiratory pressure ≥ 20cmH_2_O, positive end-expiratory effort ≥ 6cmH_2_O, arterial partial pressure/fractional inspired oxygen ratio ≤ 200 and absence of cough being the variables most associated with failure. Thus, the weighted total score showed good discriminatory and predictive power for predicting failure in planned extubation.
